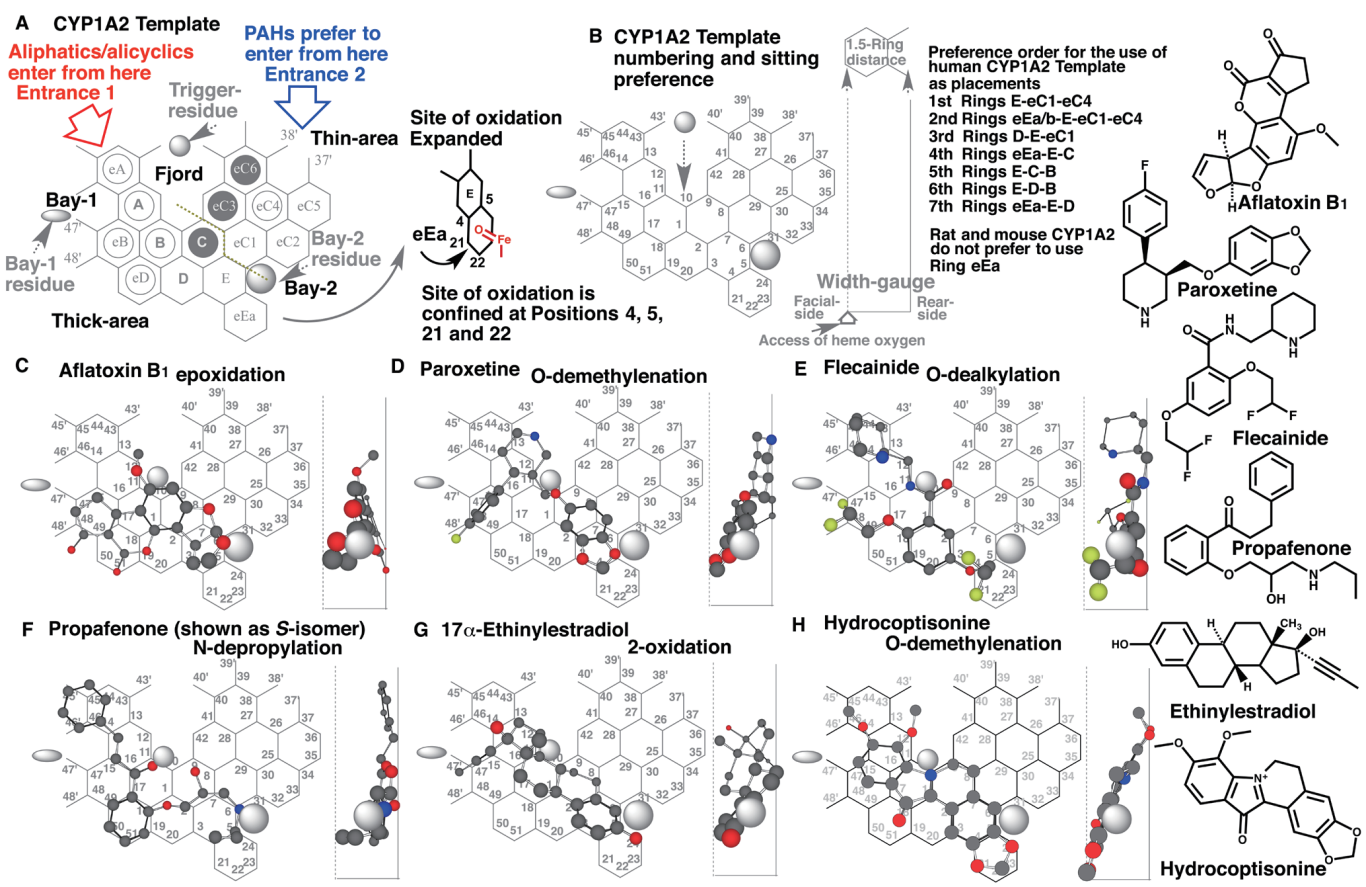# Application of CYP1A2-Template System to Understand Metabolic Processes in the Safety Assessment

**DOI:** 10.14252/foodsafetyfscj.D-22-00008

**Published:** 2022-12-23

**Authors:** Norie Murayama, Takashi Yamada, Yasushi Yamazoe

**Affiliations:** 1 Showa Pharmaceutical University, Machida, Tokyo 194-8543, Japan; 2 Division of Risk Assessment, Center for Biological Safety Research, National Institute of Health Sciences, 3-25-26 Tonomachi, Kawasaki-ku, Kawasaki 210-9501, Japan; 3 Division of Drug Metabolism and Molecular Toxicology, Graduate School of Pharmaceutical Sciences, Tohoku University, 6-3 Aramaki-Aoba, Aoba-ku, Sendai 980-8578, Japan

**Keywords:** updated CYP1A2 Template system, ligand-enzyme interaction, interpretation of metabolism, reactive intermediates, and inhibitory mechanism, trigger-residue.

## Abstract

Cytochrome P450 (CYP)-mediated metabolisms of four chemicals have been investigated to understand their unresolved phenomena of their metabolisms using human CYP-Template systems developed in our previous studies (Drug Metab Pharmacokinet 2019, 2021, 2022). Simulation experiments of a topoisomerase-targeting agent, amonafide, offered a possible new inhibitory-mechanism as Trigger-residue inactivation on human CYP1A2 Template. *N*-Acetylamonafide as well as amonafide would inactivate CYP1A2 through the interference of Trigger-residue movement with their dimethylaminoethyl parts. The mechanism was also supported on the inhibition/inactivation of two other drugs, DSP-1053 and binimetinib. Both the drugs, after other CYP-mediated slight structural alterations, were expected to interact with Trigger-residue for the intense inhibition on CYP1A2 Template. Possible formation of reactive intermediates of amonafide and 3-methylindole was also examined on CYP1A2 Template. Placements of amonafide suggested the scare *N*-oxidation of the arylamine part due to the Trigger-residue interaction. Placements of 3-methylindole suggested the formation of a reactive intermediate, 3-methyleneindolenine, rather selectively on rodent CYP1A2 than on human CYP1A2, in consistent with the experimental data. These results suggest that CYP Template systems developed are effective tools to warn an appearance of unstable reactive intermediates. Our CYP-Template systems would support confident judgements in safety assessments through offering the mechanistic understandings of the metabolism.

## 1. Introduction

Cytochrome P450 (CYP) is involved in the oxidative and reductive metabolisms of wide variety of hydrophobic chemicals. These biological processes often link to the efficacy and adverse events of the chemicals. To predict CYP-mediated metabolisms of these chemicals, 3D-models derived from crystalized CYP enzymes have been developed. The prediction is, still, difficult tasks for most of ligands taking various conformations.

Considerable amounts of experimental data on ligand-interactions of CYP enzymes have been accumulated in three-decades through the uses of the recombinant enzymes. The properties of each specific enzyme are accessible in the published materials. With the use of these advantages, we have been developing *in silico* systems to understand CYP-mediated metabolisms by the ways of the reconstitutions of ligand-accessible spaces through assemblies of CYP ligands and also of the understanding of modes of interactions of CYP-residues with ligands in the active site. Contiguous hexagonal-grid Templates* were constructed for several CYP enzymes. Template systems combined with ideas of ligand-interacting modes were established on CYP1A1(>350)^1^^)^, CYP1A2(>450)^2^^)^, CYP2C9(>500)^3^^)^, CYP2C19(>450)^4^^)^, CYP2E1(>340)^5^^)^, CYP3A4^6^^,^^7^^)^, CYP3A5^7^^)^ and CYP3A7 (>1,100 with CYP3As)^7^^)^ through reciprocal comparison of simulation and experimental results (numbers of reactions examined are shown in parentheses). Placements of ligands on Template systems of human CYP1A1, CYP1A2, CYP2C9, CYP2C19, CYP2E1 and CYP3As offered the information on sites of metabolisms regio- and stereo-selectively with more than 99% of accuracies. These Template systems are shown as effective tools for drug metabolism prediction and safety assessment^8^^,^^9^^,^^10^^)^.

One of the advantages of our Template systems is deciphering of the possible formation of unstable metabolites like reactive intermediates. Understandings of the chemical mechanism of the metabolite productions offer valuable information for the safety assessments. In the present study, unique CYP1A2-associated phenomena have been studied to understand the interaction mechanisms of CYP1A2 with ligands using the CYP1A2 Template system updated recently.

## 2. Materials and Methods

### 2.1 3D-structure Construction

Chem3D (version 5 for Mac OS, CambridgeSoft, Cambridge, MA), ChemBio3D (version 12 for Windows, CambridgeSoft), and ChemBioDraw (versions 11 and 13 for Mac OS, CambridgeSoft/PerkinElmer) were used to construct two-dimensional (2D) and three-dimensional (3D) structures of the substrates, and also to overlay compounds on Template. Substrates, except for polyaromatic hydrocarbons (PAHs), take various conformations due to their flexibility. Prior to the Template application, chemicals are taken in their flattened forms. The flatted or extended shapes of 3D structures were tried to sit on Template, and then modified their conformations to fit within Template in consideration of the bond-energy barrier using MM2 function of Chem3D and of specific interactions at distinct regions of Template. Ligands were assumed to migrate from Entrance to Site of oxidation without changing the conformation. Thus, ligands enter Template with the same conformations observed at the Site of oxidation. Chemicals including lactone moieties are often ionized at neutral pH ranges. These lactones were treated as ionizable groups for the application of substrates throughout our CYP Template systems^3^^,^^5^^,^^11^^)^. Thus, non-rigid lactone rings are not allowed to contact with Bay-2 residue and Trigger-residue of CYP1A2 Template in general.

The placement of ligands is expressed in a hyphen-linked form, such as Rings A-B-C, to trace the occupancy of chemical molecules on Template. The branching part is indicated in the bracket. Carbon, oxygen, nitrogen, sulfur, fluorine and bromine atoms of 3D ligand structures are indicated with gray, red, blue, yellow, khaki, and brown symbols, respectively. The hydrogen atoms of the substrates were not considered for the placement. To avoid the confusion from stereo and Ring indications, italic symbols are used for chemical elements like *N* and *C* in the text, but not in the figures.

### 2.2 Template Systems

Ligands were applied on specific CYP Templates following published protocols for CYP1A1^1^^)^, CYP2C9^3^^)^, CYP2C19^4^^)^, CYP2E1^5^^)^ and CYP3A4^7^^)^. Renewed CYP1A2 Template was used in the present study (Supplement Fig. 1A and B). Width-gauge was introduced in the renewed CYP1A2 system (Supplement Fig. 1B), instead of Ring eEb, Template 3 and Template 4 in the previous CYP1A2 Template system^12^^,^^13^^)^. Placements of typical ligands, such as aflatoxin B1^14^^)^, paroxetine^15^^)^, flecainide^16^^)^, propafenone^17^^)^, 17α-ethinylestradiol^18^^)^ and hydrocoptisonine^19^^)^ were generated on renewed CYP1A2 Template (Supplement Fig. 1C-H). These were constructed previously on the Template system including Ring eEb, Template 3 and Template 4. Various shapes of CYP1A2 ligands were found to be applicable in a mutual way on renewed CYP1A2 Template. Distance between Facial- and Rear-walls was determined arbitrarily as 1.5 Ring diameter from the simulation results of several CYP1A2 ligands. Procedure and rule described below are not changed from the previous system reported elsewhere^12^^,^^13^^)^.

**Fig. 1.**
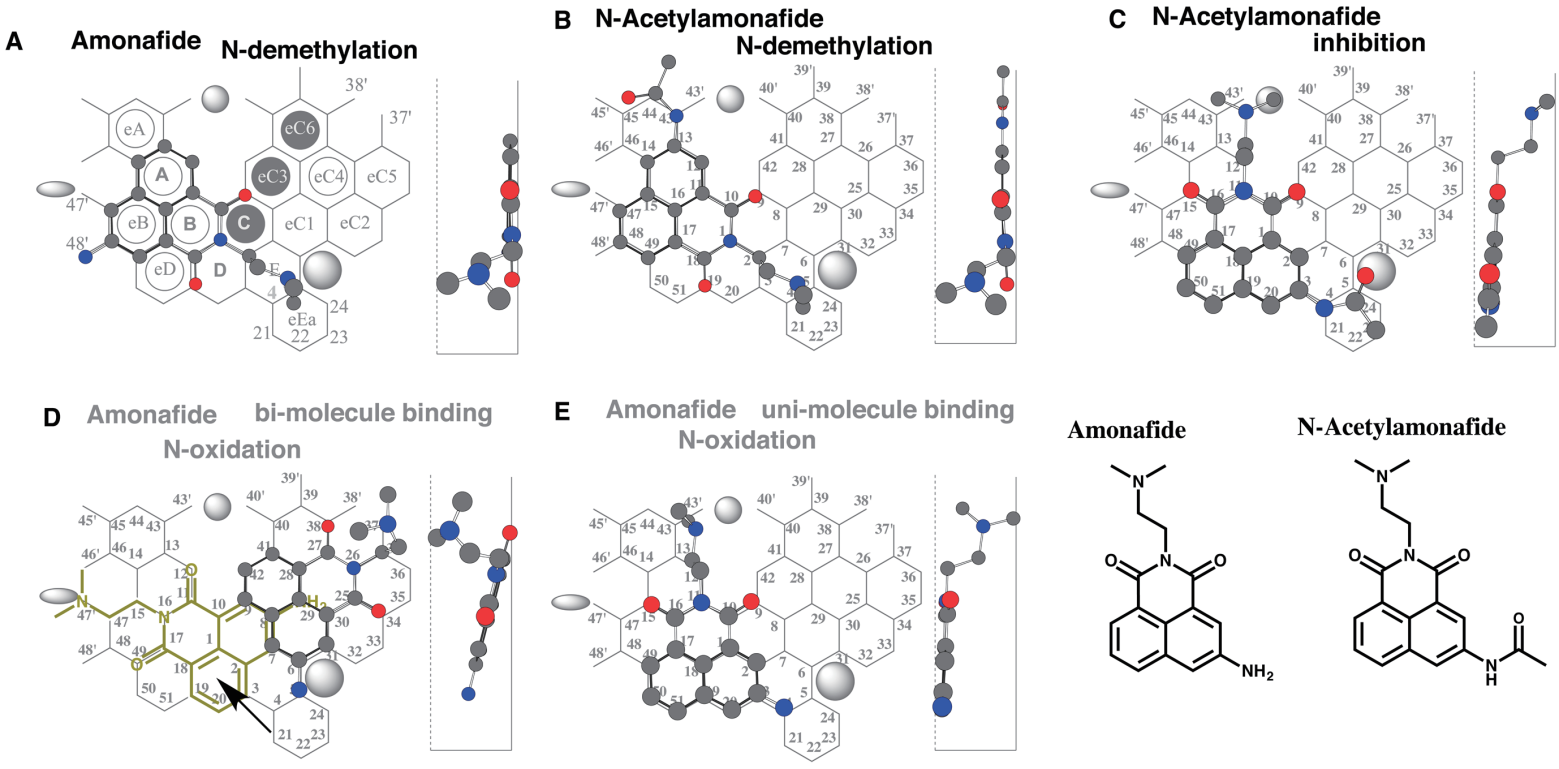
Placements of amonafide *N*-demethylation (A), *N*-acetylamonadife *N*-demethylation (B), and inhibition (C), amonafide *N*-oxidations bi-molecule binding (D) and uni-molecule binding (E) are shown as cylindrical shapes of 3D structures on CYP1A2 Template. 2D structures of amonafide and *N*-acetylamonafide are also shown in the bottom right.

### 2.3 General Rule for Application of Ligands on CYP1A2 Template

Good substrates of CYP1A2 satisfy three essential occupancies/contacts at Position 10/11 (Trigger-site), Position 9 (Facial-side pushing) and Position 4/5 or Position 21/22 (Site of oxidation) as uni-molecule or bi-molecule binding^13^^)^. Idea of bi-molecule binding is introduced to explain the regioselective metabolisms on Template. On the simulation of bi-molecule bindings, two molecules are distinguished as pro-metabolized and trigger molecules. Pro-metabolized molecule is the substrate to be oxidized or reduced, whereas Trigger-molecule is not oxidized but acts for triggering the catalysis at Trigger-site. Trigger molecule thus replenishes the essential trigger-site occupancy on Template. Trigger molecule needs to have an overlapping region with the pro-metabolized molecule on Template, but not necessarily a direct contact. Trigger molecule supports the immobilization of pro-metabolized molecule in ways to sit behind of the pro-metabolized molecule. Sittings of trigger molecules are restricted in Trigger-molecule harboring area shown as open and closed circles (Supplement Fig. 1A). Overlaps of pro-metabolized and trigger molecules are allowed on Rings C, eC3 and eC6 (Overlapping-area, closed circles)^13^^)^.

Ligands enter from Entrance-1 and/or Entrance-2 and migrate to Position 4/5 or Position 21/22 (Site of oxidation for aryl compounds) (Supplement Fig. 1A right). Ligand passage is limited with Bay-1 residue and Trigger-residue, and with Trigger-residue and Bay-2 residue. PAHs and other thin-shape ligands prefer to take placements using Rings eC1-6 (Supplement Fig. 1B right), possibly because of the ease to be held at this relatively thin-width area^13^^)^. This right-side area is thus termed Thin-area. Relative frequencies of placement usages on Thin-area are indicated for human CYP1A2^13^^)^ (Supplement Fig. 1B right). A distinct preference was observed on rodent (mouse and rat) CYP1A2 Template^2^^)^. Ligands on rodent CYP1A2 Template prefer to use Ring D, instead of Ring eEa.

The aliphatic and alicyclic ligands are unable to pass through Thin-area. These ligands thus pass through the left-side area termed Thick-area. Relatively bulky ligands such as aliphatic and alicyclic ligands as well as PAHs are also accommodated in Thick-area. Migrations of ligands from Thick-area to Thin-area are restricted and seldom to occur at Overlapping area^2^^)^. The border line between Thick- and Thin-areas is indicated as a gray dotted line (Supplement Fig. 1A). Sittings ligands at Ring eEa are restricted, but PAHs and its derivatives may slide down to Ring eEa particularly on human CYP1A2 Template (Supplement Fig. 1H). The oxidation occurs at Position 21/22, instead of Position 4/5, suggesting the access of the heme bound oxygen-atom from the Facial-side around Ring eEa^13^^)^ (Supplement Fig. 1A right). Ligands fulfilling three essential occupancies/contacts are immobilized through the descending of Trigger-residue and contact with Bay-2 residue (Supplement Fig. 1B). Trigger-residue would thus initiate the catalytic reaction.

## 3. Results

### 3.1 Interaction Mechanisms of Amonafide and the Metabolite with CYP Enzymes

Amonafide is a topoisomerase-targeting agent acting through the drug-stabilized of DNA cleavable complex formation, although the development as a chemotherapeutic drug was discontinued^20^^)^. This chemical is distinct from other arenes having primary amine group, because of the non-detectable formation of the *N*-oxidation in biological systems. Amonafide is metabolized to the *N*-demethylated and the *N*-oxide of *N,N*-dimethylaminoethyl part in dogs deficient *N*-acetylating capacity^21^^)^. In humans, *N*-acetylation of the primary amino group takes place together with the *N*-demethylation and *N-*glucuronidation^22^^)^.

Genetic difference in a *N*-acetyltransferase, NAT2, is associated with the safety of this agent. Patients who are fast acetylators of amonafide have increased toxicity at standard doses of amonafide^23^^,^^24^^)^. Furthermore, the estimated area under the plasma concentration-time curve of amonafide was significantly greater in the fast acetylators, indicating that the total plasma clearance was rather lower in this group^25^^)^. This paradoxical phenomenon has been considered to be attributable to competition for oxidation of amonafide with its acetylated metabolite^25^^)^. The mechanisms of the competition of the oxidation and of the lack of the *N*-oxidation, however, remain obscure.

Therefore, possible involvements of hepatic CYP enzymes were examined using several of our CYP Template systems developed^1^^,^^2^^,^^3^^,^^4^^,^^5^^,^^7^^,^^11^^,^^26^^)^. The 7-oxidation of amonafide was expected on CYP3A4 Template at Rings B-A-D-K-Q’(Q) plus Positions 25 and 9’ (data not shown), although the amonafide molecule would pass barely the gate of Bay-1 and Cavity-2. In consistent, a mono-hydroxylated metabolite of amonafide was isolated in dog excreta^21^^)^. A placement for the *N*-demethylation was constructed on CYP2C9 Template at Rings J(M)-I-D(H)-C-B plus a space around Position 19 (data not shown), but the *N*-dimethylamino part might be not fastened well like the case of phenylbutazone^3^^)^. A placement for the 5,6-oxidation was generated on CYP1A1 Template at Rings eEc-eC2-eC1-C-eC3 for the pro-metabolized molecule) and at Rings eA-A-B-eD-D plus Positions 9 and 4 for the trigger-molecule (data not shown). No significant levels of CYP1A1 are, however, expressed in human livers^27^^,^^28^^)^. No feasible placements were available on Templates of CYP2C19 and CYP2E1. Thus, amonafide molecule was next applied on CYP1A2 Template. Two placements for the *N*-demethylation were available at Rings E(eEa)-D-B(C)-eB-A plus Position 48’ (Fig. 1A) or Position 43 (data not shown). Both the placements fulfilled three essential interactions at Position 10/11 (Trigger-site), Position 9 (Facial-side pushing) and Position 4/5 (Site of oxidation), suggesting the role of CYP1A2. These simulation results were consistent with a proposal on the involvement of CYP1A2 on amonafide oxidation^24^^)^. Flipping of the dimethylamino group at Ring E would also give placements for the *N*-oxide formation (data not shown).

Amonafide is extensively *N*-acetylated *in vivo* in humans. A placement of *N*-acetylamonafide for the *N*-demethylation was generated at Rings E(eEa)-D-B(C)-eB-A-eA plus Position 44’ (Fig. 1B). Terminal *N*-acetyl group at Ring eA was not immobilized and thus possible to interact with Trigger-residue at Fjord region. Another placement of *N*-acetylamonafide was generated at Rings E(eEa)-D-eD-B(C)-A-eA plus a space at Fjord (Fig. 1C). The flexible dimethylaminoethyl part was thus possible to inhibit CYP1A2 through Trigger-residue interaction.

CYP1A2-mediated oxidations initiate after the immobilization of ligands through descending of Trigger-residue to Positions 10/11 on the Template system. These results suggested that poor CYP1A2-mediated *N*-demethylation of *N*-acetylamonafide might occur through an intra-molecule interaction at plural-points at Site of oxidation and Trigger-residue.

On CYP1A2 Template, two distinct areas exist and termed Thin-area and Thick-area. Ligands having flat shapes prefer to take Thin-area than Thick-area for the approach in Template. A pro-metabolized molecule placement was constructed at Rings eC1-eC3-eC4(eC5) plus Position 5 and a space around Position 37’(Entrance-2) as the consequence of amonafide sitting in Thin-area (Fig. 1D). The primary amino group was located at Position 5 for the *N*-oxidation. Sitting of a second molecule (Trigger molecule) at Rings D(C-eC3)-B-eB-A plus a space around Position 47’ was necessary to occupy Trigger-site (Position 10/11). The dimethylaminoethyl part interfered with Bay-1 residue and exceeded the limit of Template area. In addition, trigger molecule was not allowed to stay at Ring D. This bi-molecule binding was thus not functional. Another placement for the *N*-oxidation was constructed as uni-molecule binding at Rings E-D-eD-B-eB-A plus Position 9 and a space above Ring A (Fig. 1E). This placement fulfilled three essential interactions at Position 10/11 (Trigger-site), Position 9 (Facial-side pushing) and Position 4/5 (Site of oxidation). The primary amino part at Position 4 of Ring E was expected to undergo the *N*-oxidation on Template. The *N*-oxidation was, however, not detected experimentally^21^^,^^22^^,^^25^^)^. As a cause of the poor catalysis, the dimethylaminoethyl part around Rings A-eA was possible to interact with Trigger-residue to interfere the descending in similar to the case with *N*-acetylamonafide (Fig. 1C). These results indicated a possibility of the intra-molecule trigger-residue interactions to yield the poor CYP1A2-mediated *N*-demethylation of *N*-acetylamonafide and *N*-oxidation of amonafide.

### 3.2 Inactivation of CYP1A2 after Other P450-mediated Slight Structural Alterations

A unique phenomenon of human CYP1A2 is reported with DSP-1053^29^^)^. This chemical shows a time-dependent inhibition of CYP1A2 in microsomal systems of the human liver. DSP-1053 shows CYP1A2 inhibition in the presence of both recombinant CYP1A2 and CYP3A4, although DSP-1053 does not undergo CYP1A2-mediated oxidation. Additional experiments shows that CYP3A4-dependent formation of DSP-1053 imine is responsible for the time-dependent inhibition^29^^)^. The exact mechanism, however, remains unclear for the CYP1A2 inhibition.

**Fig. 2.**
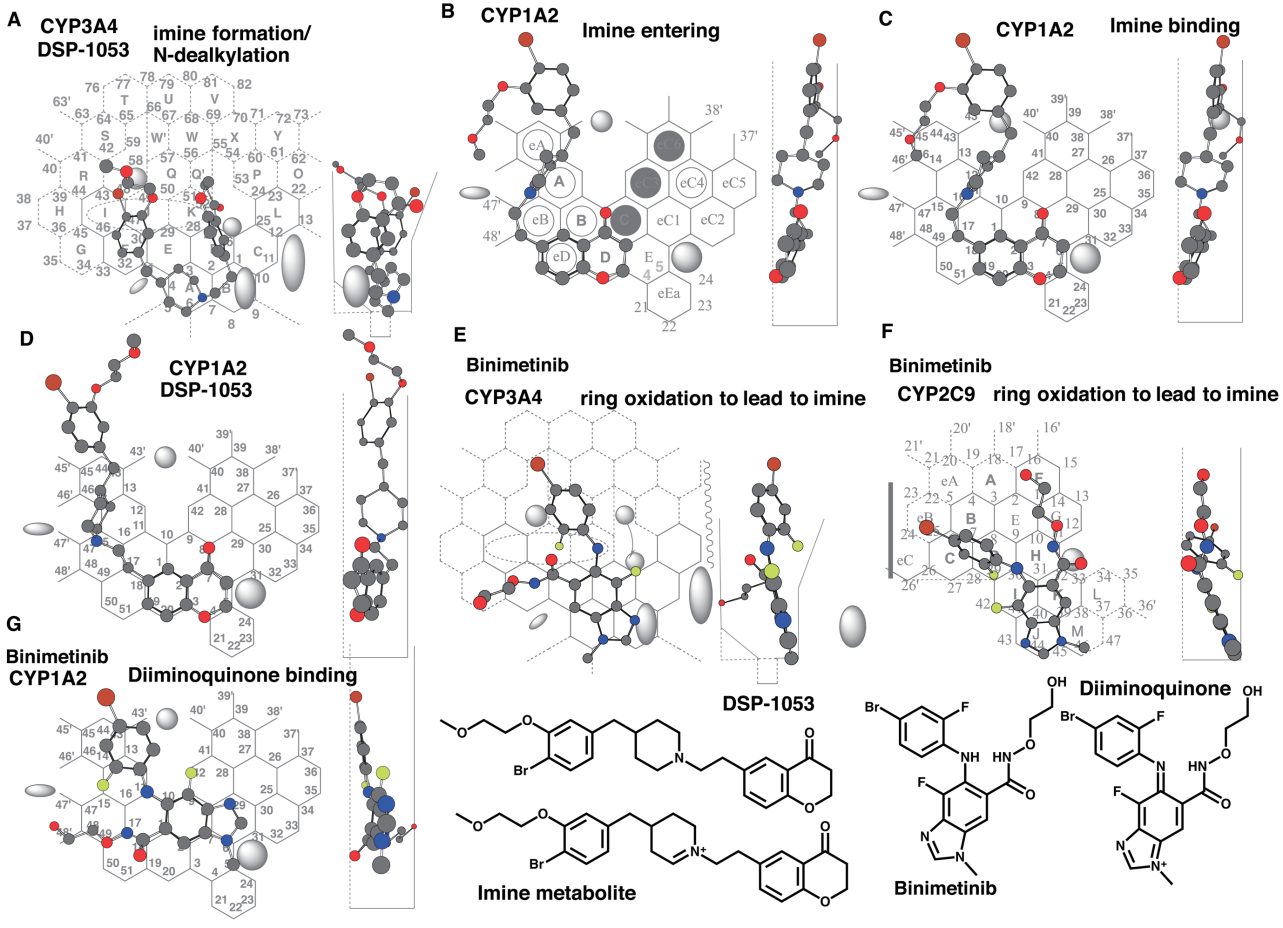
Placements of DSP-1053 imine formation and *N*-dealkylation on CYP3A4 Template (A), of imine metabolite entering (B) and sitting (C), and of DSP-1053 on CYP1A2 Template (D), of binimetinib imine formation on CYP3A4 Template (E) and CYP2C9 Template (F), and of diminoquinone metabolite on CYP1A2 Template (G) are shown as cylindrical-shape of 3D structures. 2D structures of DSP-1053, binimetinib and their imine metabolites are also shown as 2D structures.

A placement of DSP-1053 for the imine formation was available on CYP3A4 Template at Rings L(O)-C-B-A-E-K-Q(Q’)-W’ plus a space at Bay-1 (Fig. 2A). The chroman-4-one part interacted with Trigger-residue at Position 26 and with Facial-wall. The piperidine and bromobenzene parts contacted with Rear-wall, and the methoxyethyl ether part contacted with Facial-wall. This sitting satisfied three essential interactions, IJK-interaction, Trigger-residue contact, and sitting at Site of oxidation on CYP3A4 Template. These interactions were expected to support the sitting of DSP-1053 for the dehydrogenation of the piperidine ring and the cleavage of the ethyl bridge part (*N*-dealkylation) to yield M-1^29^^)^.

Sitting of the dehydrogenated metabolite (imine) on CYP1A2 Template was next examined.

This metabolite has flexible bonds and certain extent of thickness, and thus was expected to enter from Thick-area on CYP1A2 Template. A placement of the dehydrogenated metabolite was generated at Rings D(B)-eD-eB-A-eA plus a space above and left-side of Ring eA (Fig. 2B). This molecule was able to pass through a gate between Bay-1 residue and Trigger residue, and expected to move to the right-direction for the interaction of the oxygen atom of the pyrone with heme at Rings E (Fig. 2C). The resultant placement did not fulfill three essential interactions, but would yield the direct contact of the terminal ring containing bromine and methoxyethyl ether parts with Trigger-residue at Fjord area. This interaction would interfere with the descending of Trigger-residue, if this had a dissociation resistant property through stacking between Facial and Rear-walls.

A placement of a parent chemical, DSP-1053, was also constructed on CYP1A2 Template at Rings E(C)-D-B-A-eA plus a space above Ring eA (Fig. 2D). A placement of DSP-1053 taking Fjord-side sitting of the piperidine ring part was not constructed due to the steric repulsion. The allowable placement (Fig. 2D) was unlikely to interact with Trigger-residue and thus released without difficulty, suggesting that DSP-1053 was a poor substrate of CYP1A2.

Binimetinib undergoes CYP3A4- and CYP2C9-mediated oxidations to the 1,4-diiminoquinone derivative^30^^)^, which is chemically reactive and forms a glutathione adduct. Placements for the 1,4-diiminoquinone formation on CYP3A4 Template and on CYP2C9-Template were available at Rings B(A)-D(C)-K(J-I-G)-Q’(Q)-W (Fig. 2E), and at Rings J(M)-K(H-G-E-F)-I-D-C-eB (Fig. 2F), respectively. The 1,4-diiminoquinone formation, assessed as the glutathione adduct, is only in trace in recombinant CYP1A2 system^30^^)^. The 1,4-diiminoquinone, however, inactivates CYP1A2.

A placement of the 1,4-diiminoquinone was generated on CYP1A2 Template at Rings E-eC1-C(eC3)-B(eB)-A-eA plus spaces around Position 48’ and near Trigger-residue at Fjord (Fig. 2G). This placement fulfilled three essential interactions at Positions 10/11 (Trigger-site), Position 9 (Facial-side pushing) and Position 5 (Site of oxidation), and suggested occurrences of two distinct phenomena. One was the activation of the 1,4-diiminoquinone through the methyl oxidation at Site of oxidation (Position 5). The other was a possible trapping of descended Trigger-residue through the fluorine part after flipping of the 2-fluoro-4-bromo-phenyl part. The prolonged stay of Trigger-residue at Site of oxidation might support the inactivation of CYP1A2.

Experiments of both DSP-1053-derived and binimetinib-derived imines for CYP1A2 interactions supported the idea of inhibitory interaction at Trigger-residue, proposed with amonafide (Section 3.1 Interaction mechanisms of amonafide and the metabolite with CYP enzymes).

### 3.3 Distinct Substrate Specificities of Rodent and Human CYP1A2 with 3-methylindole

Although a common name, CYP1A2, is used for both the rodent (rat and mouse) and human enzymes, differences are observed on their catalytic properties, particularly on their regioselective metabolisms of PAHs^31^^,^^32^^)^. In our previous CYP1A2-Template study^2^^)^, these differences were explained as the distinct use of Ring eEa on rodent (rat and mouse) and human CYP1A2 Templates for sittings of PAHs such as benzo[c]phenanthrene and phenanthrene. Both rodent and human CYP1A2A ligands prefer a placement at Rings E-eC1-eC4, if the placement is available. The next preferred placement includes Ring eEa such as Rings eEa-E-eC1-eC4 on human CYP1A2, while ligands prefer Rings D-E-eC1 and does not use placements including Ring eEa on rodent CYP1A2 Template. Experiments on CYP1A2 Template with arylamine ligands including 2-amino-3-methyl-9H-pyrido[2,3-b]indole (MeAαC), 2-amino-3,8-dimethylimidazo[4,5-f]quinoxaline (MeIQx) and 2-n-propylquinoline also supported the idea of distinct use of Ring eEa^2^^)^.

**Fig. 3.**
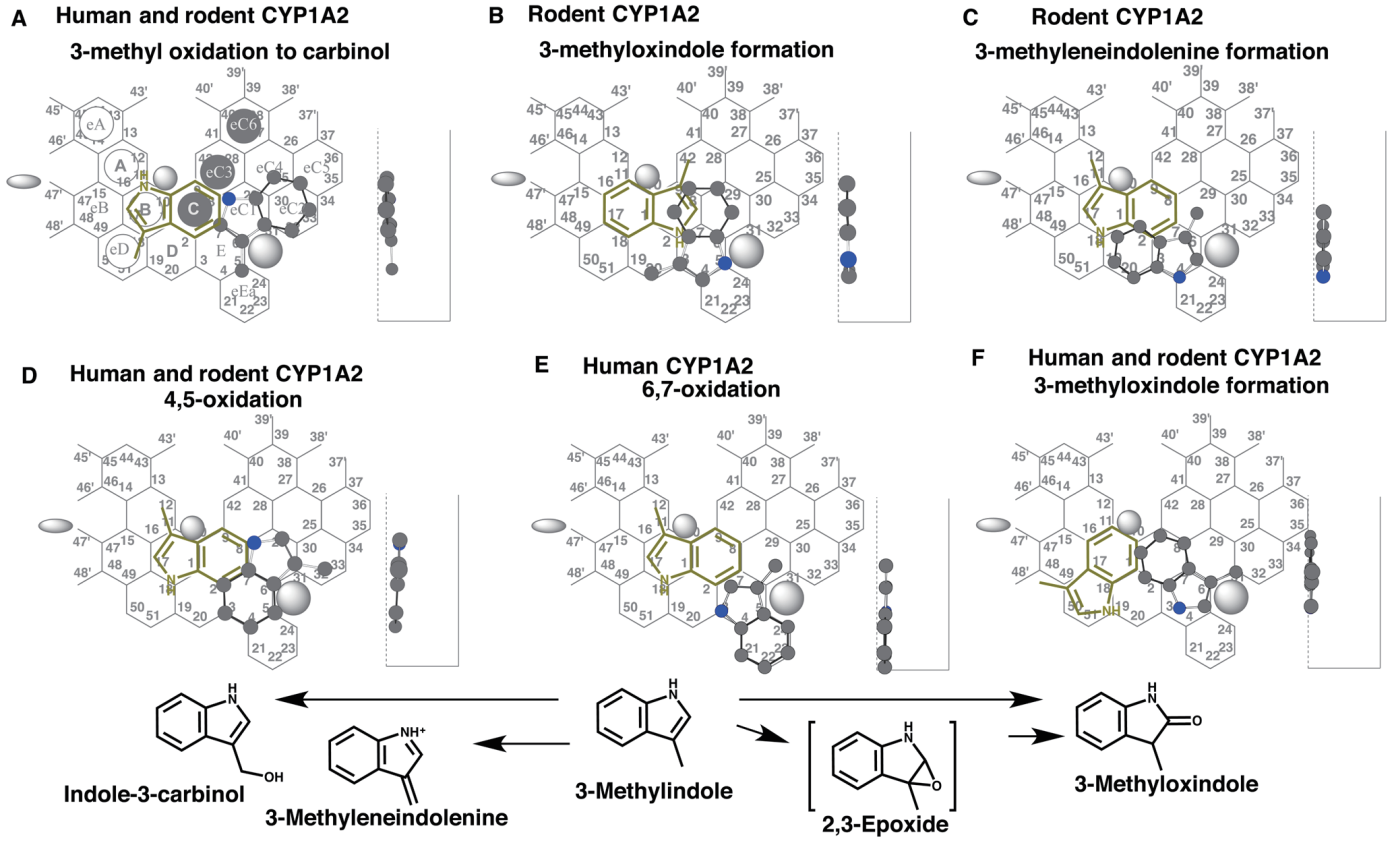
Placements of 3-methylindole for the 3-methyl oxidation (A), for the 2-oxidation (B), for the 3-methyleneindolenine formation (C), for the 4,5-oxidation (D), for the 6,7-oxidation (E), and for the 2-oxidation (F) are shown as bi-molecule binding. Trigger molecules are shown as yellow-green 2D-structures. Pro-metabolized molecules are shown as cylindrical-shapes of 3D-structures. 2D structures of 3-methylindole and its metabolites are also shown as 2D structures at the bottom. A possible non-functional placement (F) is shown as gray-color name.

3-Methylindole (skatole) is associated with acute bovine pulmonary edema and interstitial emphysema^33^^)^. The increased dietary protein, specifically the amino acid tryptophan in the diet, provides substrates for a two-step conversion to 3-methylindole through ruminal microflora-mediated deamination and decarboxylation^34^^)^. This pulmonary toxicant is metabolized, through epoxidation and dehydrogenation pathways, to indole-3-carbinol, 3-methyloxindole and 3-methyleneindolenine (Fig. 3). 3-Methyleneindolenine is believed to be responsible for the 3-methylindole toxicity^35^^)^. Several P450 enzymes, CYP1A1, CYP1A2, CYP1B1, CYP2E1, and CYP2F1, mediate these reactions^36^^)^. A strike difference is reported on the irreversible binding of ^14^*C*-3-methylindole. The ^14^*C*-binding was clearly detected in the presence of mouse CYP1A2 but only marginally in the presence of human CYP1A2^37^^)^.

A placement of 3-methylindole for indole-3-carbinol formation was available as bi-molecule binding at Rings E-eC1-eC2 (pro-metabolized molecule) and at Rings C-B-eD (trigger molecule) (Fig. 3A). Both rodent and human CYP1A2 would prefer this placement, judged from the placement usage.

Pro-metabolized molecule placement for the formation of 3-methyloxindole and also possibly the 2,3-epoxide was available at Rings D-E-eC1(C) (Fig. 3B). In addition, a distinct placement for the formation of 3-methyleneindolenine was generated at Rings D-E-eC1 (pro-metabolized molecule) and at Rings C-B-A (trigger molecule) (Fig. 3C). Ring D sitting was detected for the placements of 3-methyloxindole and 3-methyleneindolenine. These simulation results suggested the role of rodent-CYP1A2 on the formation of the reactive intermediates of 3-methylindole. These simulation results were consistent with the data of ^14^*C*-3-methylindole experiment described above^37^^)^. Rodent CYP1A2 might use the placements of 3-methylindole (Figs. 3A-C) following the preference order established with PAH ligands (Supplement Fig. 1B right).

The 4,5-oxidation of 3-methylindole was expected from a placement of the pro-metabolized molecule at Rings E-eC3-eC2 (Fig. 3D). Both human and rodent CYP1A2 would catalyze this reaction. A distinct placement of 3-methylindole was generated at Rings eEa-E-eC1 for the 6,7-oxidation (Fig. 3E). Human CYP1A2 would use preferably this placement. In addition, sitting of 3-methylindole at Rings E-C plus Position 32 was constructed for the formation of 3-methyloxindole for both human and rodent CYP1A2 after the slight rotation of the molecule anticlockwise (Fig. 3F). Of course, the migration of 3-methylindole molecule to Site of oxidation from Entrance-1 (upper left side) was possible for the pro-metabolized molecule, but was expected to be minimal judged from the experimentally established preference order for the use of human CYP1A2 Template (Supplement Fig. 1).

In consistent with the present simulation results, the 4,5- and 6,7-oxidized metabolites of 3-methylindole were detected in hepatic microsomal system of human origin^38^^)^. These simulation results were again consistent with profiles of species difference on CYP1A2-mediated metabolic activation of 3-methylindole^37^^)^.

## 4. Discussion

Precise identifications of metabolic processes are necessary to understand the safety of chemicals in humans. To verify the role of CYP1A2 on three distinct phenomena, modes of interactions of CYP1A2 have been investigated with a fused-grid based Template system of CYP1A2 in the present study.

Poor substrates of CYP1A2 become the inhibitors or inactivators after other CYP-mediated slight structural alterations. The inhibitory actions of DSP-1053 was undetectable in individual recombinant CYP1A2 systems^29^^)^. Bioactivation of binimetinib, assessed with formation of the GSH adduct, was detected in the presence of CYP2C9 and CYP3A4, but only in trace in the presence of CYP1A2^30^^)^. Clear inhibitory phenomena of both metabolites to CYP1A2 were explained as the interference of Trigger-residue function on Template (3.2 Inactivation of CYP1A2 after other P450-mediated slight structural alterations). The interaction with Trigger-residue was also observed on the placement of *N*-acetylamonafide (3.1 Interaction mechanisms of amonafide and the metabolite with CYP enzymes). Although the inhibition had been explained as the consequence of the competitive inhibition of *N*-acetylamonafide on amonafide metabolism^25^^)^, the present simulation study suggests the mechanism of CYP1A2 inactivation through the interaction of *N*-acetylamonafide with Trigger-residue prior to the descending. Tight contact of the dimethylaminoethyl part of *N*-acetylamonafide with Trigger-residue at upper Fjord region is expected for the suppression of CYP1A2 function.

On drug interactions involving oral contraceptive formulations containing 17α-ethinylestradiol, oral contraceptives markedly increase plasma concentrations and effects of tizanidine through the inhibition of CYP1A2^39^^)^. The area under the plasma concentration versus time curve (AUC) of CYP1A2-substrate, melatonin, is increased (~5-fold), and the 6-hydroxymelatonin/melatonin AUC ratio is decreased (88%)^40^^)^. 17α-ethinylestradiol, however, showed only a modest inhibition in recombinant CYP1A2 system^41^^)^. Rodrigues and colleagues noted that “17α-ethinylestradiol continues to be an enigmatic drug from the viewpoint of P450 drug interactions”^41^^)^.

CYP1A2 mediates the 2-oxidation of 17α-ethinylestradiol^18^^)^. A placement of 17α-ethinylestradiol for the 2-oxidation was available at Rings E(eEa)-C-B-A-eB plus a space around Position 47’ (Supplement Fig. 1G). The 18-methyl group of 17α-ethinylestradiol is expected to keep in contact with descended Trigger-residue.

In the present study, a new inhibitory mechanism of CYP1A2, interaction with Trigger-residue, has emerged with the use of CYP1A2 Template system (Figs. 1 and 2, and Supplement Fig. 1G). Only few examples are available at present for Trigger-residue-mediated inhibitions, and thus accumulations of events and knowledges associated with Trigger-residue would be necessary to define this phenomenon. These results on inhibitions hence support the idea of Trigger-residue involvement on CYP1A2 interaction with ligands proposed in our previous studies^2^^,^^12^^,^^13^^)^.

Detection of reactive intermediates is a purpose of metabolic studies of chemicals. CYP enzymes are involved often in this metabolic process. Their detections are performed with the use of selective and sensitive devices at present, but are still time-consuming and difficult tasks. Possibilities of detections of reactive intermediates such as *N*-hydroxylamine, arene oxide and diiminoquinone were examined on CYP Template systems (Figs. 1D, 2G and 3D). The results obtained in the present and previous studies^1^^,^^2^^,^^8^^,^^9^^)^ suggest our CYP Template systems as effective tools to warn an appearance of unstable reactive intermediates. Our CYP-Template systems would support confident judgements in safety assessments through offering the mechanistic understandings of the metabolism.

## Supplementary materials

**Figure:**